# DMSO-Free Processing of Tin–Lead Perovskite Thin Films for Solar Cells with Enhanced Stability

**DOI:** 10.1021/acsenergylett.5c03416

**Published:** 2026-01-23

**Authors:** Isabella Poli, Mirko Prato, Hui Li, Cecilia D. Costa, Luca Gregori, Daniele Meggiolaro, Tristan Quinson, Angelica Chiodoni, Filippo De Angelis, Annamaria Petrozza

**Affiliations:** † Center for Sustainable Future Technologies, 121451 Istituto Italiano di Tecnologia, via Livorno 60, Torino, 10144, Italy; ‡ Materials Characterization Facility, 121451 Istituto Italiano di Tecnologia, Via Morego 30, Genova, 16163, Italy; § Center for Nano Science and Technology, 121451 Istituto Italiano di Tecnologia, Via R. Rubattino 81, Milan, 20134, Italy; ∥ Department of Chemistry, Biology and Biotechnology, 9309 University of Perugia, INSTM, Via Elce di Sotto 8, Perugia, 06123, Italy; ⊥ Computational Laboratory for Hybrid/Organic Photovoltaics (CLHYO), 9327 Istituto CNR di Scienze e Tecnologie Chimiche “Giulio Natta” (CNR-SCITEC), Via Elce di Sotto 8, Perugia, 06123, Italy; # SKKU Institute of Energy Science and Technology (SIEST), Sungkyunkwan University, Suwon 440-746, South Korea

## Abstract

The stability of Sn–Pb perovskite semiconductors thin films remains a major challenge for their integration into efficient and durable photovoltaic devices. In this work, we demonstrate that the incorporation of methylammonium chloride (MACl) in DMSO-free Sn–Pb precursor solutions significantly enhances both the structural and operational stability of Sn–Pb perovskite films and solar cells. MACl-processed films exhibit enhanced crystallinity, environmental stability and photostability, thus tackling the most critical instabilities related to the defect chemistry of tin in tin-based perovskites and halides in lead-based perovskites. We show that Cl^–^ preferentially resides at the Pb_0.5_Sn_0.5_I-terminated surface, reducing the formation probability of halide interstitials, preventing I_2_ loss under illumination and reducing O_2_ uptake under ambient air exposition. As a result, solar cells incorporating MACl-treated films maintain stable performance under maximum power point tracking for over 900 h. This work highlights the crucial role of interfaces and paves the way for more durable perovskite solar cells.

## Graphical abstract

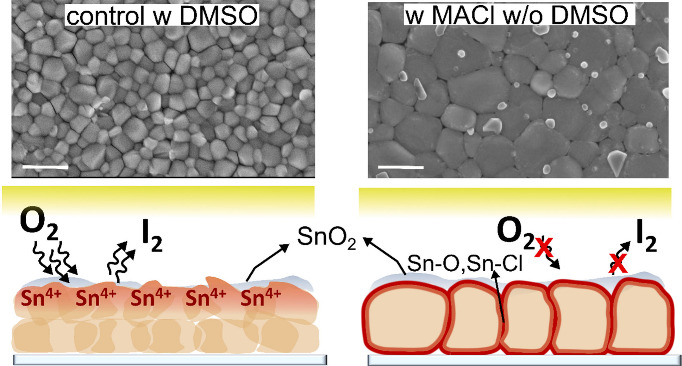

Sn–Pb mixed halide perovskite (HP) is a promising composition for both efficient single junction solar cells and tandem applications due to their optimal bandgap that can be narrowed down to 1.25 eV.^1^ Despite this potential, the practical efficiencies achieved in single-junction solar cells still fall below the radiative efficiency limit of over 30%,^2^ with the current highest recorded power conversion efficiency (PCE) of approximately 24%.^3,4^ Furthermore, challenges in reproducibility persist, as it remains difficult to consistently achieve efficiencies above 20%, with many reports showing PCEs in the range of 15–20%.^5,6^ The limiting factors to both reproducibility and efficiency are the high sensitivity of Sn­(II) to oxidation,^7^ and the different crystallization rates of Sn and Pb, which cause inhomogeneities.^8,9^

The most used fabrication route in the HP field consists of a N,N-dimethylformamide/dimethyl sulfoxide (DMF/DMSO) solvent system with an antisolvent step to quench and control the perovskite growth, and form smooth, pinhole-free films.^10–12^ This procedure has been adopted regardless material composition and high-performance Pb, Sn and Sn–Pb perovskites have been demonstrated with such fabrication route. However, recent studies highlight potential issues with the use of DMSO in Sn and Sn–Pb halide perovskites. First, DMSO can contribute to Sn oxidation.^13–18^ and, given its high boiling point, it can get trapped within the film causing instabilities.^19,20^ Furthermore, it was recently demonstrated that Sn­(II) and Pb­(II) exhibit different interactions with donor ligands, with lower cohesive energy in SnI_2_–DMSO than in PbI_2_–DMSO complexes.^21^ The different crystallization rates of Sn and Pb, together with their distinct coordination to DMSO, can induce the formation of nonuniform films with heterogeneous distribution of Sn and Pb, particularly near the upper surface. Achieving similar crystallization rates for Sn and Pb leads to more homogeneous elemental distribution, reduced energetic disorder and higher open circuit voltages.^9^

A promising approach to reduce these inhomogeneities is to explore DMSO-free systems. DMF alone results in nonuniform films, negatively impacting device performance, therefore a crystallization agent is necessarily required.^22^ McMeekin et al. introduced a DMSO-free processing method that uses dimethylammonium chloride (DMACl) as an additive to control the growth of a formamidinium (FA)/cesium (FA)_*y*_Cs_1–*y*_Pb­(I_*x*_Br_1–*x*_)_3_ perovskite system, enabling larger grain size and improved orientation.^23^ Other works added similar quantities of methylammonium chloride (MACl) to FAPbI_3_ and FAPbBr_3_ perovskites using typical DMF/DMSO solvent systems and found enhanced grain size, film quality, and structural stability upon addition.^24–26^ Interestingly, while MACl significantly impacts the film formation process, the latter studies have reported little to no residual chloride in the final films, suggesting its role as a transient but crucial agent in the crystallization pathway.

In this study, we remove DMSO as cosolvent in Sn–Pb perovskites and use instead MACl for the first time. We find that adding MACl in DMF-based Sn–Pb precursor solutions significantly enhances the crystallinity and enlarges the grains. A combination of X-ray photoelectron spectroscopy (XPS), energy dispersive X-ray (EDX) and density functional theory (DFT) calculations suggest that chloride is retained in the film and preferentially incorporates at the surface, playing a key role in making the surface less reactive to oxidation and in suppressing iodine expulsion under illumination. As a result, solar cells fabricated using MACl-treated films demonstrate exceptional operational stability, surpassing 900 h of maximum power point tracking (MPPT) under continuous illumination.

We fabricated FA_0.85_Cs_0.15_Sn_0.5_Pb_0.5_I_3_ perovskite thin films by spin coating, comparing two preparation routes: a control system using DMF:DMSO, and a DMSO-free method using only DMF with addition of MACl. All films were prepared inside a N_2_-filled glovebox using the gas quenching technique (details in the Supporting Information). Figure 1a shows top-view Scanning Electron Microscope (SEM) images of the control DMSO-containing film and a DMSO-free film with MACl (MACl-treated). The incorporation of MACl notably increases grain size, producing smoother and more compact films. Figures S1–S3 in the SI shows the top-view SEM images of DMSO-free films with different MACl concentrations. Films processed without DMSO and without MACl exhibit poor morphology, incomplete coverage, and rough surfaces, due to the lack of stable intermediate complexes. Films prepared with 15 mol % MACl show a high density of pinholes, whereas all concentrations ≥30 mol % produce compact, continuous and pinhole-free films (Figure S1). Cross-sectional SEM images (Figure S4) reveal uniform thicknesses (approximately 500 nm) and vertically aligned grains for MACl concentrations ≥30 mol %. Previous works on pure Pb systems showed that a similar increase in grain size occurs when MACl is incorporated into the precursor solution using DMF:DMSO solvent systems^24,25,29^ or even when introduced via postprocessing to Pb-based systems.^30^ However, in this study we deliberately adopt a DMSO-free formulation to avoid DMSO promoted oxidation of Sn^2+^ to Sn^4+^.^13,31,32^

**Figure 1:**
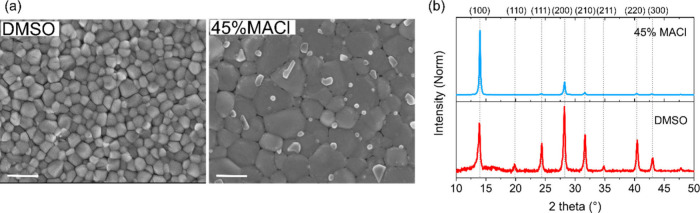
(a) Top view SEM images of mixed Sn-Pb halide perovskite thin films processed with and without DMSO and MACl as additive. Scale bar is 1 μm. (b) XRD patterns of mixed Sn-Pb halide perovskite thin films with and without DMSO and 45% MACl. The patterns have been normalized with respect to the most intense peak. The reference patterns have been taken from ref 33.

UV–visible absorption spectra in Figure S5 show absorption onsets at about 960 nm for all compositions, and the film containing 60 mol % MACl exhibits reduced absorption in the near-UV region compared with the other films. Figure S6 presents the values from the peak of the first derivative of the absorption spectra of films processed without and with MACl, showing that the addition of MACl leads to a slight narrowing of the bandgap of about 10 meV. A comparison of the X-ray diffraction (XRD) patterns of the thin films (Figure 1b and Figure S7) reveals that MACl enhances crystallinity, as evidenced by sharper and more intense diffraction peaks. The most significant improvement, characterized by the narrowest full width half-maximum (fwhm) and highest peak intensity, is observed at 45 mol % (Figure S8). Additionally, films containing MACl exhibit a preferred orientation along the (100) direction, consistent with previous observations in Pb-halide and mixed halide perovskite systems.^27^ A closer examination of the (100) peak (Figures S8 and S9) shows a shift toward higher angles with increasing MACl content, suggesting a contraction of the crystal lattice. This shift likely results from partial MA incorporation into the A-site, given the observed narrowing of the bandgap,^28^ rather than Cl incorporation, which would instead cause a bandgap widening.^27^ Furthermore, increased MA content may lead to a compositional imbalance at the A-site, which could promote Cs-rich phase segregation, aligning with the SEM and EDX results (see Figures S3 and S4 in the SI).

Considering the UV–vis, SEM and XRD results, we identified 45 mol % MACl as the optimal composition and selected it for further study and optimization. To assess the film composition, we performed SEM-EDX analysis. EDX results yield Sn-Pb atomic ratios of 1.25 and 1.2 for DMSO-containing and MACl-treated films, respectively (average values calculated over three spot areas: 1.25 +/− 0.08 and 1.13+/− 0.09), while I:(Sn + Pb) atomic ratios of 2.9 and 2.7 are obtained for DMSO-containing and MACl-treated films, respectively (average values: 2.8 ± 0.1 and 2.7 ± 0.02), closely matching the expected stoichiometric values (1.1 and 3), indicating preservation of the bulk composition (Table 1). The analysis of the MACl-treated sample also revealed the presence of Cl within the film, with a Cl:(Pb + Sn) ratio of 0.25, roughly half of what was expected based on the used amounts of MACl. While this observation confirms a relevant Cl loss in the film formation process, at the same time, it is in stark contrast to most Pb-based systems, where the formation of volatile Cl species lead to almost no Cl residual in the film.. The higher Lewis acidity of Sn^2+^ compared to Pb^2+^ likely enhances its interaction with Cl^–^, leading to its stabilization in the film.

**Table 1:** Comparison of Quantitative Results Obtained via XPS and SEM-EDX for DMSO-Containing and DMSO-Free Perovskite Films

	**Control DMSO-containing perovskite**		**DMSO-free,** 45 mol % MACl perovskite	
	**XPS**	**SEM-EDX**	**XPS**	**SEM-EDX**
O	37.8 at. %	28.3 at. %	31.0 at. %	27.7 at. %
Cl	0 at. %	0 at. %	12.5 at. %	4.4 at. %
Cs	0.4 at. %	2.4 at. %	1.2 at. %	1.7 at. %
Sn	31.9 at. %	9.8 at. %	23.9 at. %	9.8 at. %
Pb	4.7 at. %	7.8 at. %	7.2 at. %	8.1 at. %
I	25.3 at. %	51.8 at. %	24.2 at. %	48.3 at. %
Sn:Pb	6.79	1.25	3.32	1.20
I:(Sn + Pb)	0.69	2.94	0.78	2.70
Cs:(Sn + Pb)	0.01	0.13	0.04	0.09
Cl:(Sn + Pb)	0.00	0.00	0.40	0.25

To further investigate the composition and surface chemistry of the films, we performed XPS on both a DMSO-containing film and a DMSO-free film with 45 mol % MACl. Figure 2 shows the low-resolution wide-scan XPS spectra of the films and the high-resolution spectra of individual elemental regions. From the wide scan (Figure 2a), we estimated the surface chemical composition, reported in Table S1, and compared to the SEM-EDX results in Table 1.

**Figure 2:**
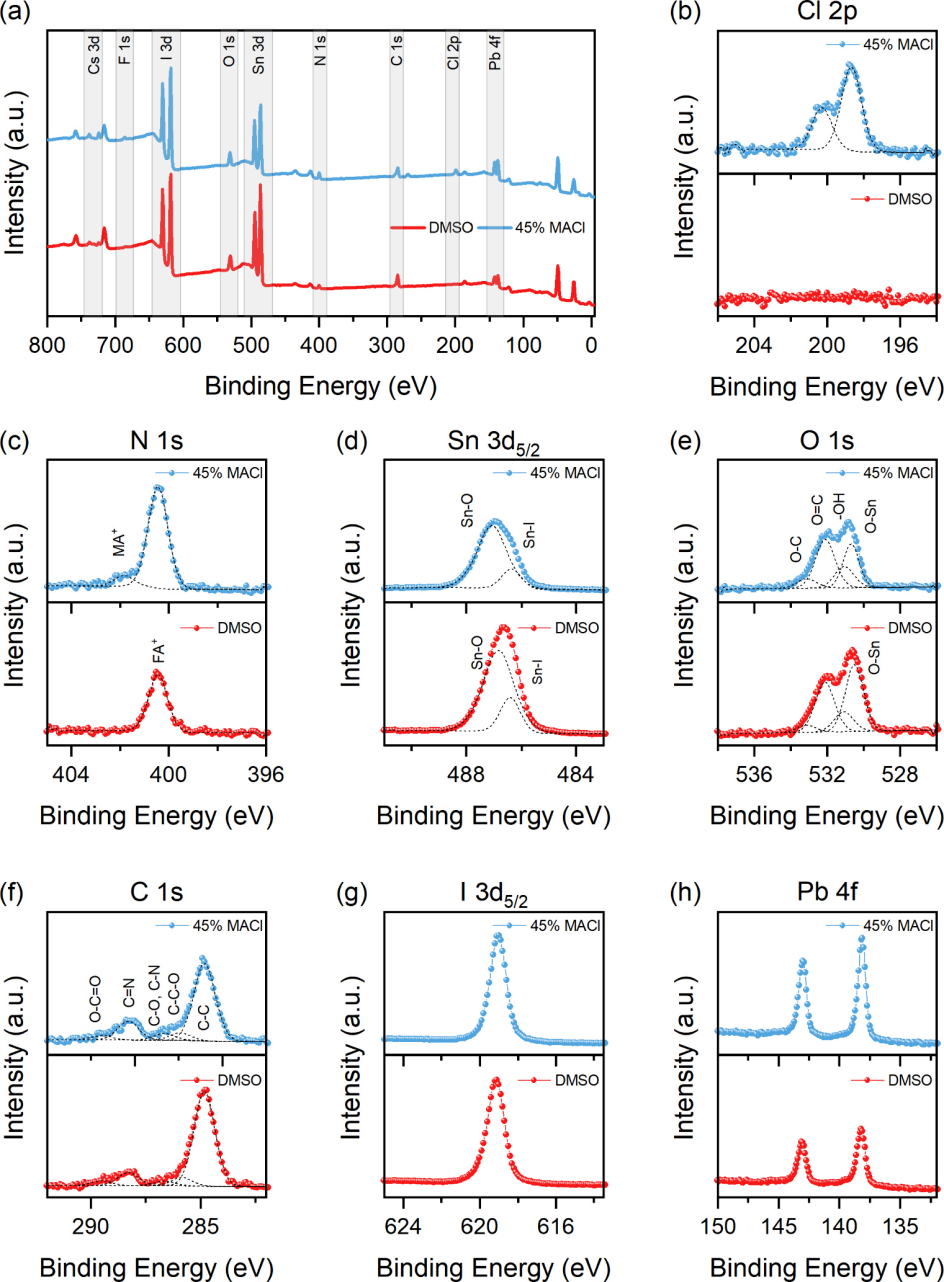
(a) Wide-scan XPS spectra. XPS spectra of the (b) Cl 2p, (c) N 1s, (d) Sn 3d_5/2_, (e) O 1s, (f) C 1s, (g) I 3d_5/2_ and (h) Pb 4f of mixed Sn-Pb perovskite thin films processed with DMSO and with 45 mol % MACl. The results of the best fit procedure are reported only for those spectra where peak positions could not be trivially desumed. The corresponding fitting parameters are listed in Table S2 in the SI.

It is relevant to highlight that the results of the XPS quantitative analysis are obtained assuming a homogeneous distribution of the observed elements within the probed volume (sampling depth ∼10 nm), and might not, therefore, represent the real sample composition. Regarding EDX, a standardless microanalysis was used; while reliable and sufficiently accurate, it should not be interpreted as providing the exact quantitative composition of the sample. The comparison between XPS and SEM-EDX results indicates that, for both samples, the top surface of the perovskite films is characterized by a strong Sn:Pb unbalance due to a large Sn excess. This is accompanied by relatively low Cs and I content. Notably, oxygen is detected on the surface of both films. This is commonly observed in Sn-containing films due to the high sensitivity of Sn^2+^ toward oxidation.^34,35^ Consistent with our previous findings on pure Sn perovskites,^34^ we find that oxygen forms multiple species at the surface of the Sn–Pb film as well. These include Sn—O bonds and C—O/C=O/H—O species associated with adventitious carbon (panel (e) of Figure 2). Another key observation is the significant surface retention of chlorine in the MACl-processed film, in line with the SEM-EDX outcome.

The N 1s spectrum of the MACl-processed film (panel (c) of Figure 2) reveals the presence of MA on its surface, indicated by the C-NH_2_ peak at 401.8 ± 0.2 eV, together with the C=NH^2+^ one from FA centered at 400.4 ± 0.2 eV. The MA peak is absent in the DMSO-processed film. Quantitative analysis of nitrogen from MA and chlorine shows a strong excess of Cl (N:Cl = 1:5.4) at the surface, suggesting that Cl^–^ does not remain bound to MA^+^, which would otherwise yield a 1:1 ratio. Instead, it appears that Cl^–^ forms separate species during film formation, while a portion of MA^+^ incorporates into the perovskite lattice. This is further supported by XRD data, which show a contraction of the perovskite unit cell upon addition of MACl.

Turning to the Sn 3d_5/2_ region (data shown in panel (d) of Figure 2), we emphasize that fitting XPS spectra of Sn-containing perovskites is challenging due to overlapping contributions from the perovskite phase (Sn^2+^) and various byproducts (SnO, SnO_2_, SnI_4_, FA_2_SnI_4_).^34^ Nevertheless, the MACl-processed film shows a broader peak with a maximum at a higher binding energy than what is observed on the control DMSO-processed case (487.0 vs 486.7 eV, respectively), suggesting the formation of different Sn species upon MACl incorporation. A recent study on CsSnI_3_ found that when SnCl_2_ was introduced in the precursor solution as an additive, it did not integrate into the perovskite lattice but formed a protective surface layer.^36^ Given the presence of oxygen and the reactivity of Sn with O_2_, we propose that tin oxychloride species form at the surface/grain boundaries. Due to the complex surface chemistry of the film, it is difficult to derive the precise composition, and we therefore refer to it generally as Sn–O and Sn–Cl bonds, acknowledging that Sn may exist in both 2+ and 4+ oxidation states. As shown in Figure 2d, we attribute the observed signals to Sn in the perovskite matrix (Sn–I component centered at 486.4 eV) and to the Sn–O component (appearing at 486.8 eV for the control DMSO-processed sample, and at 487.1 eV for the MACl-processed one), while the Sn–Cl component in the MACl-processed sample is expected to overlap to the latter.^36^ The mixed coordination of Sn with both Cl^–^ and O^2–^ might explain the observed shift to higher binding energies of the Sn–O peak and the broader Sn peak compared to the Cl-free material. Supporting this assignment, a recent study developing SnOCl as transport material for Sn–Pb perovskites reported XPS features at similar energy values.^37^ Based on the above results, we conclude that a portion of the surface Sn is bound to Cl and to O. Considering an average Sn:Cl ratio of 1, the remaining Sn can be attributed to the perovskite phase. This yields an estimated Sn/Pb ratio of approximately 1.6, which is reasonably close to the expected value of 1.1.

To evaluate whether the presence of Sn–O and/or Sn–Cl bonds on the surface affect the band-edge energetics, we measured the valence band and Fermi level positions using ultraviolet photoelectron spectroscopy (UPS). Figure S10 shows the UPS spectra and the resulting energy band diagrams for both perovskite films, with and without MACl. Both films exhibit surface n-doping, with Fermi levels located approximately at 0.9 and 0.7 eV above the valence band for the untreated and MACl-treated samples, respectively. This n-type surface character is primarily attributed to the presence of SnO_2_ on the surface, as previously reported in pure Sn perovskites.^34^ Notably, the MACl-treated film shows reduced electron affinity and ionization energy, indicating a decrease in n-doping relative to the untreated DMSO-containing reference.

The enhancement of grain size in the presence of MACl has often been attributed to nucleation effects.^38^ While additives in solution may influence the formation of precursor complexes, recent studies show no consistent correlation between additive-induced colloidal structures and final film morphology.^30^ Instead, a key factor appears to be the ability of MACl to enhance ion transport across grain boundaries during annealing, thereby promoting coarsening-driven grain growth. To understand the effect of different solvent systems and possible coordination with SnI_2_ and MACl precursors we performed ^1^H NMR and UV–vis analysis of precursor solutions, focusing on SnI_2_ in DMF, SnI_2_ in DMF:DMSO and SnI_2_ + MACl in DMF. ^1^H NMR results reveal coordination of both SnI_2_ and MACl with DMF (Figure S11). UV–vis spectra of the diluted precursor solutions in acetonitrile further demonstrates that MA^+^/Cl^–^ interacts also with SnI_2_, significantly lowering the concentration of Sn^4+^ species, probably due to the formation of strong Sn–Cl bonds already in solution (Figure S12).

We then studied the incorporation of chloride ions into the Pb–Sn perovskite by DFT simulations. The thermodynamics of substitution of the iodide ions by chloride has been evaluated in the bulk and at the Pb_0.5_Sn_0.5_I-terminated surface of the FA_0.75_Cs_0.25_Pb_0.5_Sn_0.5_I_3_ phase. The calculated energies reveal a favorable incorporation in the bulk and at the surface of FA_0.75_Cs_0.25_Pb_0.5_Sn_0.5_I_3_ phase, with values of −0.15 and −0.27 eV (Table 2). The sensibly lower energy of substitution at the surface with respect to the bulk highlights that Cl^–^ are preferentially incorporated at the surface. This result is in agreement with XPS and EDX measurements, revealing high concentrations of Cl at the surface.

**Table 2:** DFT Calculated Incorporation Energies of a Cl Ion Substituting I in the Bulk and at the Pb_0.5_Sn_0.5_I-Terminated Surface of the FA_0.75_Cs_0.25_Pb_0.5_Sn_0.5_I_3_ Perovskite^a^

	**FA**_**0.75**_ **Cs**_**0.25**_ **Pb**_**0.5**_ **Sn**_**0.5**_ **I**_**3**_
Bulk	–0.15
Surface	–0.27

a All values are in eV and are calculated at the PBE-D3 level of theory.

To assess the protective role of these surface species against air exposure, we conducted XPS on both control and MACl-treated films after they have been left for 2 days in ambient air (Figure 3a). In the DMSO-containing sample, the Sn 3d_5/2_ peak shifts significantly to higher binding energies, indicating complete oxidation of Sn^2+^ in the perovskite phase to Sn^4+^. In contrast, the Sn 3d_5/2_ peaks of the MACl-treated sample do not evidence the formation of Sn^4+^ species; instead, a minor shift to lower binding energies is observed, suggesting better preservation of the perovskite phase. The oxygen signal also evolves upon air exposure. In the control DMSO-containing sample, the Sn–O peak disappears, while peaks associated with OH, O=C, and O—C bonds become more prominent, consistent with previous findings.^22^ In contrast, the sample with MACl exhibits a distinct Sn—O peak after air exposure, supporting a higher surface chemical stability. Quantification of the oxygen content (Figure S13) reveals that the control film adsorbs significantly more oxygen (35 at. %) than the MACl-treated one (15 at. %), which instead shows a similar level to the unaged sample. This suggests that the MACl addition makes the Sn surface less reactive toward O_2_, thereby reducing oxygen uptake and mitigating degradation.

**Figure 3:**
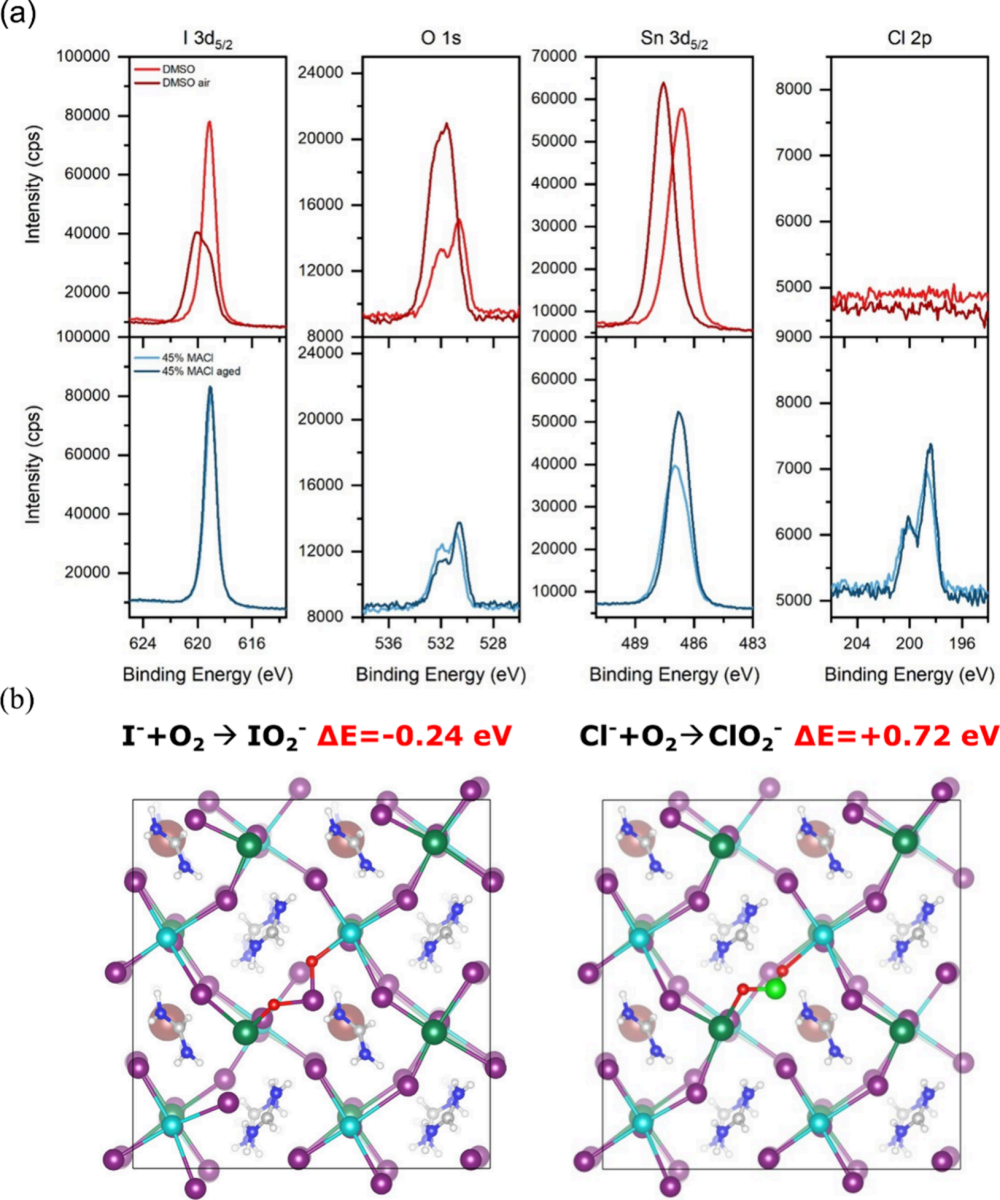
(a) XPS spectra of the I 3d_5/2_, Cl 2p, Sn 3d_5/2_ and O 1s of mixed SnPb perovskite thin films processed with DMSO (red) and with 45 mol % MACl (blue) before and after air exposure for 2 days. (b) Top view of the O_2_ dissociation with formation of IO_2_^–^ and ClO_2_^–^ structures at the Pb_0.5_Sn_0.5_I-terminated surface of FA_0.75_Cs_0.25_Pb_0.5_Sn_0.5_I_3_. In the structures, iodine atoms are shown in purple, chlorine in light green, lead in cyan, tin in dark green, cesium in red, nitrogen in blue, hydrogen in white, carbon in gray.

The protective role of Cl^–^ ions against O_2_ has been investigated by simulating the interaction of an O_2_ molecule with the pristine and Cl-doped FA_0.75_Cs_0.25_Pb_0.5_Sn_0.5_I_3_ perovskite at the Pb_0.5_Sn_0.5_I-terminated surface, see Figure 3b. Following the approach of a previous computational study,^39^ the thermodynamics of dissociation of the O_2_ molecule to form the IO_2_^–^ complex at the surface has been studied. This process simulates the first reactive step of the oxygen-induced degradation of the perovskite after the removal of the organic moiety by water.^39^ As can be observed in Figure 3b, the formation of the IO_2_^–^ complex is favored by 0.24 eV at the pristine surface, highlighting that O_2_ is progressively incorporated at the surface leading to the formation of Sn^4+^ phases and the decomposition of the perovskite. The formation of a ClO_2_^–^ complex, on the other hand, is energetically unfavorable by 0.72 eV. This is ascribed to the higher electronegativity of Cl compared to I, disfavoring lattice oxidation. These results, in combination with the evidence of a more favorable incorporation thermodynamics of Cl ions at the surface, support the hypothesis that Cl ions form stable species at the surface, making it less reactive toward O_2_.

To evaluate whether this enhanced stability also protects against photoinduced degradation, we tested the films under prolonged illumination. A known degradation route for halide perovskites involves light-driven ion migration and the formation of I_2_ at the surface, which contributes to performance loss.^40^ The photo-oxidation of the lattice to form I_2_ is the leading photodegradation process in Pb-based perovskites,^41^ even though it becomes competitive with direct tin oxidation on the low-stable surfaces of the Pb–Sn perovskites.^42^ To probe this, we compared iodine expulsion in untreated DMSO-containing control films and films treated with MACl. Both types of films were immersed in hexane-filled vials and exposed to simulated sunlight for 100 h (Figure 4a). UV–visible absorption spectra of the hexane solutions revealed distinct differences: the control sample exposed to light showed clear peaks corresponding to I_2_, while the MACl-treated samples exhibited no such signals (Figure 4b).^43,44^ Control experiments conducted in the dark confirmed that I_2_ generation is photoinduced (Figure S14). Both control and MACl-treated samples displayed absorption features near 350 nm, which can be attributed to I_3_^–^,^45,46^ suggesting that some photoinduced I_2_ is also generated in MACl-treated sample which combine with I^–^ ions to form I_3_^–^ in solution.^41^ To investigate the origin of the increased photostability, the photo-oxidation of the I sublattice has been investigated by DFT, by simulating the trapping of two photogenerated holes to form the V_I_^+^/I_i_^+^ defect couple. Simulations have been carried out in the bulk and at the surface of the pristine and Cl-doped FA_0.75_Cs_0.25_Pb_0.5_Sn_0.5_I_3_ perovskite (see Figure 4c,d). Hybrid-DFT calculations show that the formation energy of the V_I_^+^/I_i_^+^ couple is less favorable in the Cl-doped vs pristine perovskite by ∼0.2 eV, both in the bulk and at the surface. This result provides a plausible explanation for the absence of I_2_ related spectral features and the improved photostability of the MACl-treated samples.

**Figure 4:**
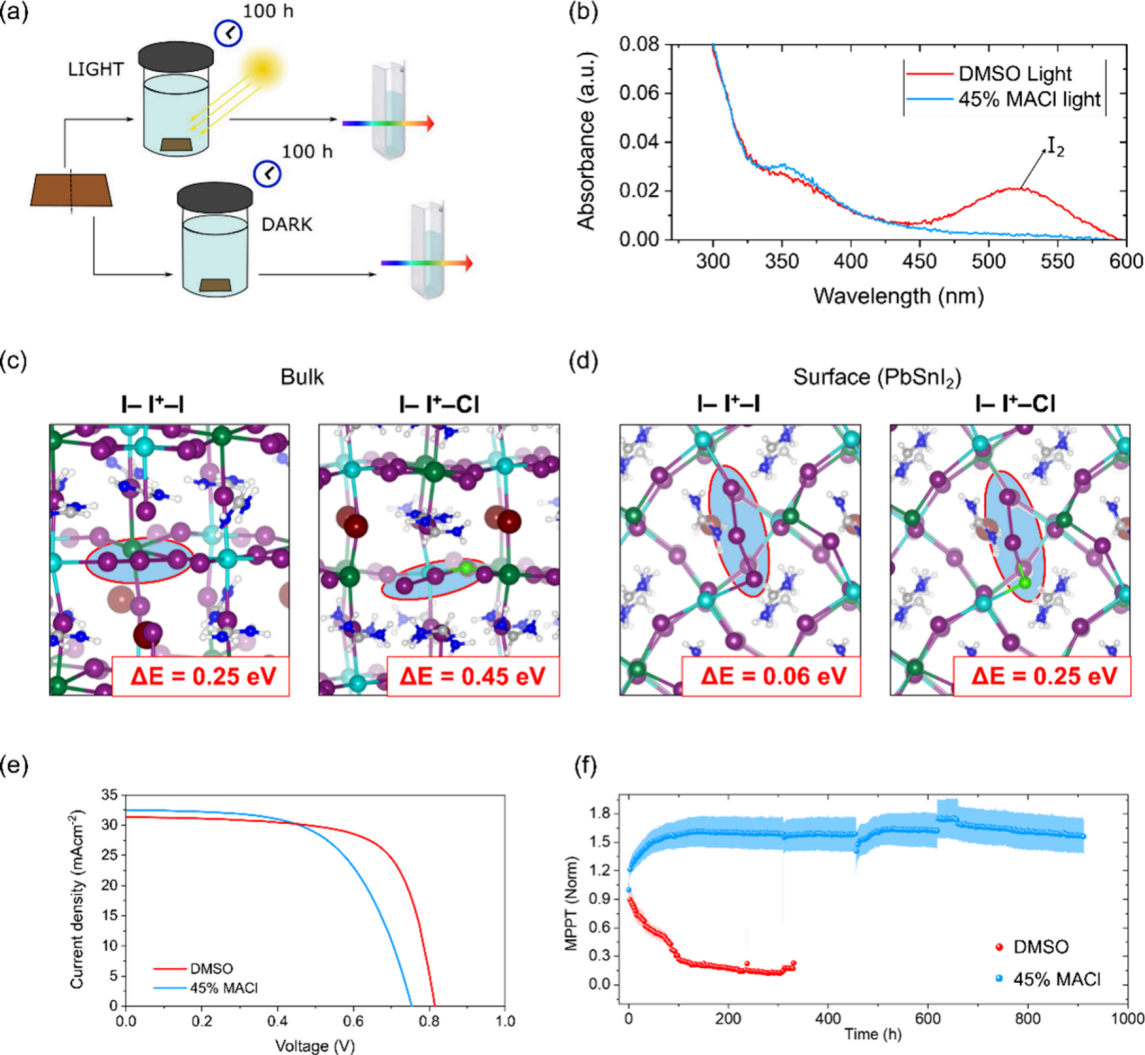
(a) Schematics of the I_2_ expulsion experiments. (b) UV–vis spectra of the hexane solution submerging the perovskite films during the I_2_ expulsion experiment after 100 h of simulated light exposure. (c) Optimized structures of the V_I_^+^/I_i_^+^ defect couple in the bulk and (d) at the PbSnI_2_ terminated surface of the FA_0.75_Cs_0.25_Pb_0.5_Sn_0.5_I_3_ perovskite with the calculated energies of formation. Iodine atoms are shown in purple, chlorine in light green, lead in cyan, tin in dark green, cesium in dark red, nitrogen in blue, hydrogen in white, carbon in gray. (e) *J*–*V* curves of the champion control DMSO-containing perovskite solar cell and the champion DMSO-free perovskite device fabricated with 45 mol % MACl. (f) MPPT stability tests of control and MACl-treated devices. solar cells have been aged at room temperature, in N_2_ under continuous MMPT and simulated 1 sun illumination. (The initial average PCE is 13.6% and 11% for the untreated and MACl-treated devices, respectively. Shaded regions represent the statistical variation across four cells of each type.)

To validate these observations, we fabricated solar cells using both untreated and MACl-treated films and tested their operational stability under maximum power point tracking (MPPT) conditions with continuous simulated sunlight at room temperature in inert N_2_ atmosphere. The current density–voltage (*J*–*V*) curves of as deposited champion devices based on control DMSO-containing cells and MACl treated Sn–Pb perovskite devices are showed in Figure 4e. The highest PCE for the control and MACl-treated samples are 17.7% and 14.6%, respectively. The statistics of PCE, open circuit voltage (*V*_OC_), short circuit current density (*J*_SC_), and fill factor (FF) for different devices are shown in Figure S15 as box plots. The average PCE for the control and MACl-treated samples are 16.2% and 12.7%, respectively. The higher fill factor observed in the control devices processed with DMSO may arise from the stronger n-type surface character compared with the MACl-treated perovskite, which can facilitate more efficient electron extraction at the perovskite/ETM interface. However, despite the control devices exhibit higher initial power conversion efficiencies, the solar cells fabricated with MACl demonstrated markedly enhanced stability compared to the control cells, when tested in N_2_ under continuous MPPT (Figure 4f). *J*–*V* curves of representative as-deposited cells are shown in Figure S16. To ensure a fair comparison, device stability was evaluated for samples with comparable PCEs and PV parameters, thereby avoiding any dependence of the MPPT behavior on the initial efficiency. The improved stability of the MACl-treated device can be attributed to the absence of DMSO as a cosolvent, which might otherwise remain trapped in the film and promote Sn^2+^ oxidation, as well as to the less favorable formation of the V_I_^+^/I_i_^+^ couple under illumination, mitigating the expulsion of I_2_. Finally, to confirm the reduced reactivity of the surface toward oxidation, we monitored the performance evolution under MPPT in ambient air without using any encapsulation (Figure S17). The devices quickly degrade, however the decay is considerably slowed down in MACl-treated samples.

In summary, we show that the addition of MACl in the precursor solution is an effective strategy to enhance the stability of Sn–Pb perovskite systems at both the material and device levels. MACl acts as a booster for improved crystallinity and morphology, leading to larger grain sizes, while also serving a passivating role, with Cl^–^ that preferentially gets incorporated at grain boundaries, specifically at the surface. This protection mitigates both oxidative and photoinduced degradation processes: on the one hand, it reduces the amount of oxygen absorbed on the surface, preserving the perovskite phase intact; on the other hand, it prevents the release of photoinduced I_2_ gas formation thanks to a reduced formation of V_I_^+^/I_i_^+^ couple. As a result, perovskite solar cells incorporating MACl exhibit outstanding operational stability, maintaining stable performance for over 900 h under continuous MPPT conditions. These findings provide valuable insights into the stabilization mechanisms of Sn–Pb perovskites and suggest a viable route toward long-lasting perovskite-based photovoltaics.

## Supplementary Material



## Notes

Experimental section, computational details and additional characterization and discussion, including SEM images, EDX, UV–vis, XRD, ^1^H NMR, XPS fitting parameters, current–density voltage curves and box plots with statistical distribution and variability of key performance PV parameters (PDF)
