# Supplementary material for: DMSO-Free Processing of Tin–Lead Perovskite Thin Films for Solar Cells with Enhanced Stability
Source: ACS Energy Lett. 2026 Jan 23;11(2):1791–9. doi: 10.1021/acsenergylett.5c03416 (PMC12910712; doi:10.1021/acsenergylett.5c03416)
Supplement: Supplementary file 1 [file nz5c03416_si_001.pdf]

Supporting Information of

DMSO-Free Processing of Tin-Lead Perovskite Thin Films for Solar Cells With Enhanced Stability

Isabella Poli,[1,*] Mirko Prato,[2] Hui Li,[3] Cecilia D. Costa,[1] Luca Gregori,[4,5] Daniele Meggiolaro,[5] Tristan Quinson,[3] Angelica Chiodoni,[1] Filippo De Angelis,[4,5,6] Annamaria Petrozza[3,*]

1. *Center for Sustainable Future Technologies, Istituto Italiano di Tecnologia, via Livorno 60, Torino , 10144 ,Italy*
2. *Materials Characterization Facility, Istituto Italiano di Tecnologia, Via Morego 30, Genova, 16163 Italy*
3. *Center for Nano Science and Technology, Istituto Italiano di Tecnologia, Via R. Rubattino 81, Milan, 20134 Italy*
4. *Department of Chemistry, Biology and Biotechnology, University of Perugia, INSTM, Via Elce di Sotto 8, Perugia, 06123, Italy*
5. *Computational Laboratory for Hybrid/Organic Photovoltaics (CLHYO), Istituto CNR di Scienze e Tecnologie Chimiche "Giulio Natta" (CNR-SCITEC), Via Elce di Sotto 8, Perugia, 06123, Italy*
6. *SKKU Institute of Energy Science and Technology (SIEST), Sungkyunkwan University, Suwon 440-746, South Korea*

1. Methods

Materials

N,N-dimethylformamide (DMF, anhydrous, 99.8%), dimethyl sulfoxide (DMSO, anhydrous, ≥99.9%), anisole (anhydrous, 99.7%), acetone (ACS reagent), isopropyl alcohol (IPA, ACS gradient), tin(II) iodide (AnhydroBeads, −10 mesh, 99.99%) , tin(II) fluoride (SnF_2- 99%) and MACl (for synthesis) were purchased from Sigma-Aldrich. PbI_2 (99.99%, for Perovskite precursor) was purchased from TCI. All chemicals were used without any further purification. Microscope slides (Thermo Scientific) were used as substrates for XPS and optical characterization and ITO-coated glass (youxuan) for solar cell fabrication. Silicon substartes were used for SEM and EDX characterization. All substrates were cleaned in diluted Hellmanex III, deionized water, acetone and isopropyl alcohol (IPA) for 10 min by sonication before deposition. The cleaned glass substrates were treated with oxygen plasma for 10 min before any further deposition. Thin-film

perovskite deposition was performed in a nitrogen-filled glovebox. Thin films used for photoluminescence (PL) measurements and solar cells were glass-encapsulated with a Masterbond UV-curable adhesive (LED401), which cures within 10 seconds under UV light exposure. This process was conducted in the glovebox to prevent oxygen exposure. The sealing ensures the material's stability for at least one month. However, for this work, all characterizations were carried out within one week after fabrication for all batches.

Sn-Pb control thin films: to make Sn-Pb based ($FA_{0.85}Cs_{0.15}Pb_{0.5}Sn_{0.5}I_3$) perovskite thin-films, the precursor solution (concentration of 1.2 M) was prepared by dissolving FAI, CsI, and SnI_2 in a mixture of DMF and DMSO solvents (volume ratio of 4:1). The molar ratio for FAI/CsI was 0.85:0.15 and the molar ratio of (FAI+CsI)/SnI_2 was 1:1. 10 mol% (relative to SnI_2) of SnF_2 was added within the precursor solution. The precursor solution was stirred at room temperature for 30 min. The perovskite films were deposited with one-step spin-coating procedure at 1000 r.p.m. for 10 sec, followed by 4000 r.p.m. for 50 s. After 17 seconds from the beginning of the spin coating, a N_2 stream centred onto the rotating substrate was flowed until the end of the spinning procedure. The substrates were annealed at 120°C for 20 min.

Sn-Pb thin films with MACl: To make MACl-tretaed Sn-Pb based ($FA_{0.85}Cs_{0.15}Pb_{0.5}Sn_{0.5}I_3$) perovskite thin-films, the precursor solution (concentration of 1.2 M) was prepared by dissolving FAI, CsI, and SnI_2 in DMF together with different quantities of MACl. The same spinning procedure of the control films and post annealing were performed.

Sn-Pb based solar cells: The patterned ITO substrates (2.8x2.8cm^2) were washed in Hellmanex 2% deionised water (DI) solution, DI water, acetone, and ethanol for 10 minutes sequentially. After that, the well-dried ITO substrates were treated in a UV plasma cleaner for 10 min. The cleaned ITO substrates are immediately transferred to N_2 filled glovebox. The devices used for MPPT stability tests consisted in PEDOT/perovskite/C60/BCP/Ag. The PEDOT precursor solution was prepared by diluting PEDOT (HTL3 from Clevios) with anhydrous toluene (1:4 v:v). The PEDOT solution was spin coated onto ITO substrates at 500 rpm for 3 seconds and 4000 rpm for 30 s, and then baked on a hotplate at 150 °C for 10 min. After annealing, the ITO/PEDOT substrates were allowed to cool down to room temperature naturally. Subsequently, a thin layer of Al_2O_3 was deposited on top of PEDOT. Al_2O_3 nanoparticles dispersion (Sigma-Aldrich) was diluted with IPA and spin-coated onto ITO/PEDOT substrates at 4500 rpm for 30 sec and annealed for 3 minutes at 100°C. The perovskite solution was prepared as detailed above (with and without MACl) and spun at 1000 rpm for 10 seconds and 4000 rpm for 40 seconds. After 17 seconds from the beginning of the spin coating, a N_2 stream centred onto the rotating substrate

was flowed until the end of the spinning procedure. The substrates were annealed at 120°C for 20 min. The film was annealed at 120 °C for 20 minutes. As electron selective contact, C60 (30 nm) and BCP (3 nm) were subsequently evaporated at a 10-6 mbar vacuum level with a deposition rate between 0.1 and 0.3 Å s-1 Finally, 100 nm of Ag was evaporated at a 10-6 mbar vacuum level with a deposition rate between of 0.1 up to 10 nm and 0.1-1 Å s-1 up to 100 nm. The devices were glass encapsulated in the glovebox before taking them out to be tested. The devices showed in Figure 4a-4b are based on 1.8 M $FA_{0.7}MA_{0.3}Pb_{0.5}Sn_{0.5}I_3$. For control device, the precursor solution was prepared by mixing 1.26 M FAI, 0.54 M MAI, 0.9 M PbI_2, 0.9 M SnI_2, 0.045 M SnF_2, and 5 mg Sn powder in co-solvent DMSO/DMF (1 mL, 1:3 volume ratio). The perovskite layer was deposited by spin-coating above precursor solution with two-step spin-coating procedures: 1000 rpm for 10s and 4000 rpm for 40 s. Chlorobenzene (400 μl) as antisolvent was rapidly dripped onto the spinning substrate over an interval of 1 s during the second spin-coating step at 10 s before the end of the procedure, then annealing at 100°C for 10 min. For target device, the precursor solution was mixing 1.26 M FAI, 0.54 M MAI, 0.9 M PbI_2, 0.9 M SnI_2, 0.045 M SnF_2, 5 mg Sn powder and 45 mol% MACl in 1 mL DMF. The perovskite solution was spun at 1000 rpm for 10 seconds and 4000 rpm for 40 seconds. After 17 seconds from the beginning of the spin coating, a N_2 stream centred onto the rotating substrate was flowed until the end of the spinning procedure. The fabricated substrates were subsequently transferred to a vacuum chamber where 25 nm C60 (deposition rate of 0.01 nm·s-1), 5 nm BCP (deposition rate of 0.01 nm·s-1) and 80 nm Au electrode were consecutively deposited by thermal evaporation on the above substrate to the devices.

Characterization

Both the sample preparation and mounting for XPS took place in a N_2-filled glovebox, ensuring an oxygen concentration below 3 ppm. Subsequently, the samples were directly transferred into the XPS chamber using a transfer chamber, ensuring that the thin films have never been exposed to air prior to XPS analysis. Instead, spectroscopy measurements have been performed on encapsulated samples.

X-ray photoelectron spectroscopy (XPS): XPS was carried out on a Kratos Axis Ultra^{DLD} spectrometer. Thanks to their high conductivity, around 10 S/cm, films deposited on glass did not experience sample charging issues during XPS measurements. Additionally, the surface of the sample was contacted with the holder through copper tape. Wide scans were acquired at a pass energy of 160 eV, with an energy step of 1 eV, over a (300 x 700) μm^2 area. High-resolution spectra were acquired at a pass energy of 10 eV and energy step of 0.1 eV, over the same

analysis area. All the spectra were obtained using a monochromatic Al K*α* source (15 kV, 20 mA). XPS data were analyzed using CasaXPS (version 2.3.24)[1] To account for possible charging effects, the C 1s peak due to the C-N bonds in formamidinium was used as internal reference for the binding energy scale, and its position was set to 288.3 eV, in agreement with literature reports.[2] UPS was carried out on the same spectrometer, using a He I (21.22 eV) discharge lamp, on an area of 55 μm in diameter, at a pass energy of 10 eV and with a dwell time of 100 ms. The work function (that is, the position of the Fermi level with respect to the vacuum level) was measured from the threshold energy for the emission of secondary electrons during He I excitation. A −9.0 V bias was applied to the sample to precisely determine the low-kinetic-energy cutoff, as discussed in ref[3]. The position of the cutoff was then estimated with CasaXPS software, using the “Edge Up” background function for the energy interval around the cutoff. Then, the position of the VBM versus the vacuum level was estimated by measuring its distance from the Fermi level, focusing on the high-kinetic energy (i.e., low-binding energy) cutoff region and using the “Edge Down” background function in CasaXPS software.

FESEM-EDX: FESEM-EDX images were obtained using a ZEISS SUPRA 40 Field Emission Scanning Electron Microscope (FESEM) equipped with an Oxford INCA Energy Dispersive X-ray (EDX) detector. An accelerating voltage of 5kV has been used for imaging, while an acceleration voltage of 18 kV has been selected for EDX spectra acquisition. A standardless microanalysis that provides quantitative compositional data by evaluating the X-ray spectrum using fundamental physical formulas and general atomic databases was used. Perovskite films were prepared on both ITO and Si substrates. Similar morphology were obtained.
*FE*SEM images showed in the SI were obtained using a MIRA3 TESCAN microscope with an accelerating voltage of 5 kV. Perovskite films were prepared on ITO substrates.

UV-Vis Absorption: absorption spectra were measured on perovskite thin films deposited on glass using a UV/VIS/NIR spectrophotometer Lambda 1050, PerkinElmer, in the wavelength range 350–1100 nm, with step size of 1 nm.

X-ray Diffraction: XRD patterns were recorded with a Bruker D8 Advance diffractometer with Bragg–Brentano geometry equipped with a Cu Kα_1 (λ = 1.54056 Å) anode, operating at 40 kV and 40 mA. All the diffraction patterns were collected at room temperature, with a step size of 0.05 degrees in symmetric scan reflection mode and a dwell time of 1 s. XRD patterns were

recorder on thin films in an inert environment by means of a Bruker airtight specimen holder with dome like x-ray transparent cap, for environmentally sensitive materials.

Iodine expulsion experiment: Two equal cuts of the same perovskite sample are placed in two vials filled with hexane (4 ml). One vial is placed under 100 mW/cm^2 illumination with illumination ranging from 0 to 2 sun equivalent using 496 LED emitters with low mismatch 390-700 nm (Arkeo Cicci Research) for 100 hours, while the other is kept in dark. We estimate the concentration of I_2 released by the sample into the solvent by measuring the absorption spectrum of the hexane taken from the vials. The presence of I_2 generates additional features between 400-600 nm in the hexane absorption spectrum. All the displayed spectra are the result of the difference between the absorption spectra of the hexane taken from the vials after the experiment and pristine hexane.

Voltage-Current measurements: The current density - voltage (J-V) characteristics of the solar cells were measured with a computer-controlled Keithley 2420 source meter in ambient air on devices previously glass encapsulated in the glovebox. The simulated Air Mass 1.5 Global (AM 1.5G) irradiance was provided with a class AAA Newport solar simulator. The light intensity was calibrated with a silicon reference cell (KG5 window) with a spectral mismatch factor of 0.99. The active area of the complete device was determined by an illumination-shadowing mask which is 0.0935 cm^2. Sn-based cells were tested by scanning between -0.1 and 1 V with a scan rate of 0.1 V/s.

Nuclear Magnetic Resonance: ^{1}H NMR spectra were collected on a 80 MHz Spinsolve (Magritek) benchtop spectrometer. Each spectrum was acquired using 32 scans, an acquisition time of 3.2 s, a repetition time of 30 s and a 90° pulse angle, with ^{13}C decoupling.

Computational details

All the DFT calculations have been carried out with Quantum Espresso 6.3 package.[4] We started by optimizing the 2x2x2 supercell for the $FA_{0.75}Cs_{0.25}PbI_3$ and $FA_{0.75}Cs_{0.25}Pb_{0.5}Sn_{0.5}I_3$ phases. Lattice parameters were optimized from experimental values, obtaining values of a = 8.773 Å, b = 9.103 Å, 12.760 Å for Pb-based and a = 8.762 Å, b = 9.039 Å, 12.660 Å for mixed Pb-Sn phase. DFT calculations were performed using norm-conserving pseudopotentials from the PseudoDojo repository,[5] explicitly including the following atomic shells: Pb (4s, 4p, 4d, 5s, 5p), I (5s, 5p), Br (4s, 4p), N and C (2s, 2p), H (1s), Yb (5s, 5p, 5d, 6s). The calculations used a wavefunction cutoff of 60 Ry and Brillouin zone sampling at the Gamma point. To further improve the accuracy of the predictions, we also performed single-point hybrid PBE0 (α = 0.25)[6] calculations on top of the

PBE-D3 optimized structures. The calculations used a wavefunction cutoff of 60 Ry and 120 Ry on the Fock operator and Brillouin zone sampling at the Gamma point. Defect formation energies (DFE) and the thermodynamic transition levels (TTL) have been calculated by using the following equations:

$$DFE\ (X^q) = E(X^q) - E(perf) - \sum_{i}^{i} n_i \mu_i + q(E_f + V) + E^q(corr) \quad (1)$$

$$\varepsilon(q/q') = \frac{DFE(D^q, E_F=0) - DFE(D^{q'}, E_F=0)}{q' - q} + \frac{E^q_{corr} - E^{q'}_{corr}}{q' - q} \quad (2)$$

where $E(X^q)$ is the energy of the supercell containing defect X, E(perf) is the energy of the non-defective system, n and μ are, respectively, the number and the chemical potentials of the species added or subtracted to the non-defective system to form a defect; q is the charge of the defect. Long-range electrostatic interactions E^q_{corr} have been corrected through the Makov-Payne scheme by using the ionic dielectric permittivity of $MAPbI_3$ (ε=24). The chemical potentials of the atomic species were set to simulate realistic growth conditions under thermodynamic stability constraints of the perovskite phases. For the full Pb-based system, we considered two regimes: Pb-rich and Pb-poor conditions. In the Pb-rich limit, the chemical potential of lead was fixed to that of bulk metallic lead, $\mu(Pb) = \mu(Pb_{bulk}) = 0.00$ eV, while the chemical potential of iodine was derived from the stability of PbI_2 as $\mu(I) = (\mu(PbI_2) - \mu(Pb_{bulk})) / 2$. In the Pb-poor case, iodine was set to its elemental limit, $\mu(I) = \mu(I_2Solid)/2 = 0.00$ eV, and the lead chemical potential was determined accordingly as $\mu(Pb) = \mu(PbI_2) - 2\mu(I)$. For the mixed Pb–Sn system, the chemical potentials were similarly chosen to ensure phase stability, taking into account the presence of both Pb and Sn halide compounds. Under Pb/Sn-rich conditions, the chemical potentials of Sn were set $\mu(Sn) = \mu(Sn_{bulk}) = 0.00$ eV, for the iodine was derived from the stability of the SnI2 and SnI4 phases: $\mu(I) = (\mu(SnI_4) - \mu(SnI_2)) / 2$, finally for the lead, we calculated the chemical potential as $\mu(Pb) = (\mu(PbI_2) - \mu(I)*2)$. In the Pb/Sn-poor regime, the iodine potential was again set to its upper bound, and the Pb and Sn potentials were chosen to maintain the stability of the mixed Pb–Sn perovskite phase as for the full lead system. Finally, to study the protective role of Cl^- ions against O_2 we simulate the interaction of an O_2 molecule with the pristine and Cl-doped $FA_{0.75}Cs_{0.25}Pb_{0.5}Sn_{0.5}I_3$ perovskite at the $Pb_{0.5}Sn_{0.5}I$-terminated, we use a 5-layer slab of the 2x2 in-plane supercell obtained from the previous optimizations.

2. Supporting Figures

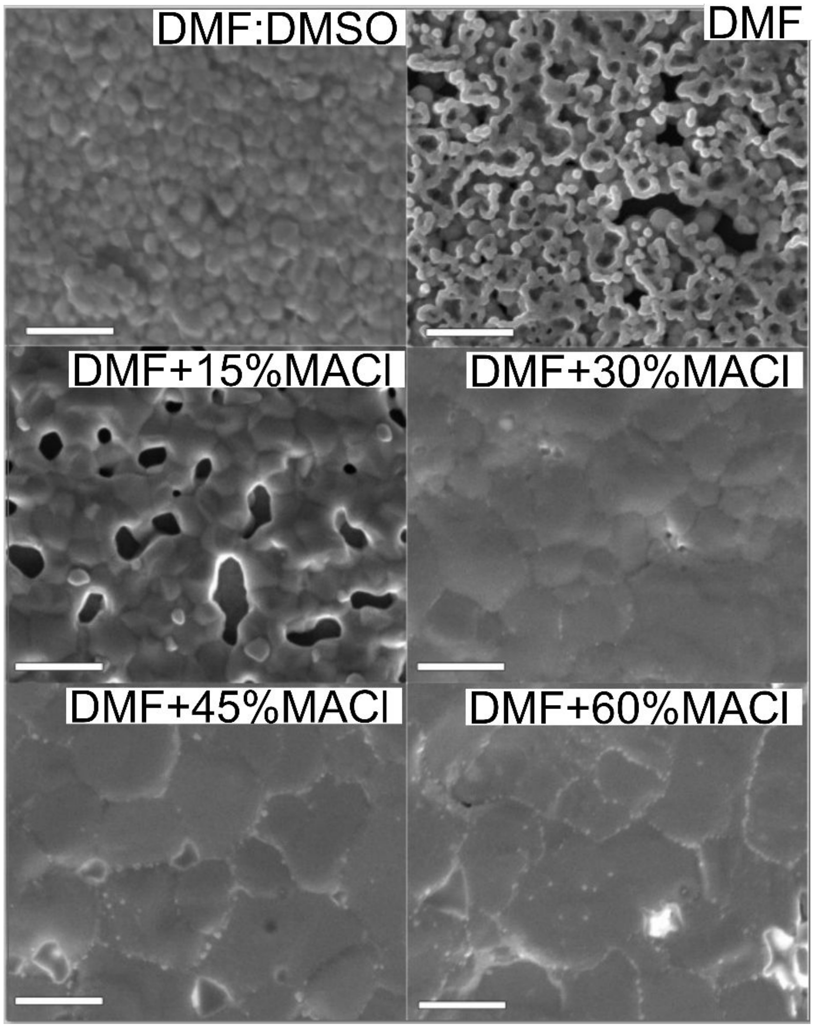

Fig. S 1. Top view SEM images of mixed SnPb halide perovskite thin films processed with and without DMSO and different quantities of MACl as additive. Scale bar is 1 μm. Adding 15 mol% of MACl improves surface coverage but results in pinholes. Compact, uniform films are obtained only at MACl concentrations above 30 mol%.

Beyond grain size, MACl addition leads to the formation of surface segregations, visible as bright spots in SEM images (Figure 1 and Figure S1). Through EDX analysis, we identify these as CsI-rich phases (Figure S3-S4). Such segregation is consistent with literature reports on Cs instability and its tendency to phase separate. [7–9]

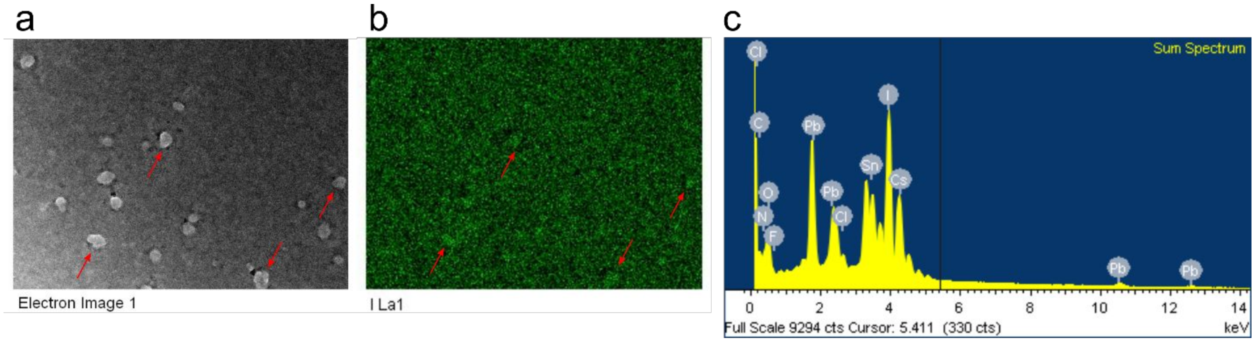

Fig. S 2. a-b. Energy dispersive X-ray spectroscopy (EDX) elemental map of Iodine of Sn-Pb halide perovskite films prepared with 45mol% MACl. Higher concentration of I are observed where brighter spots appear in the SEM top-view image. c. EDX sum spectra of the map showed in panel a.

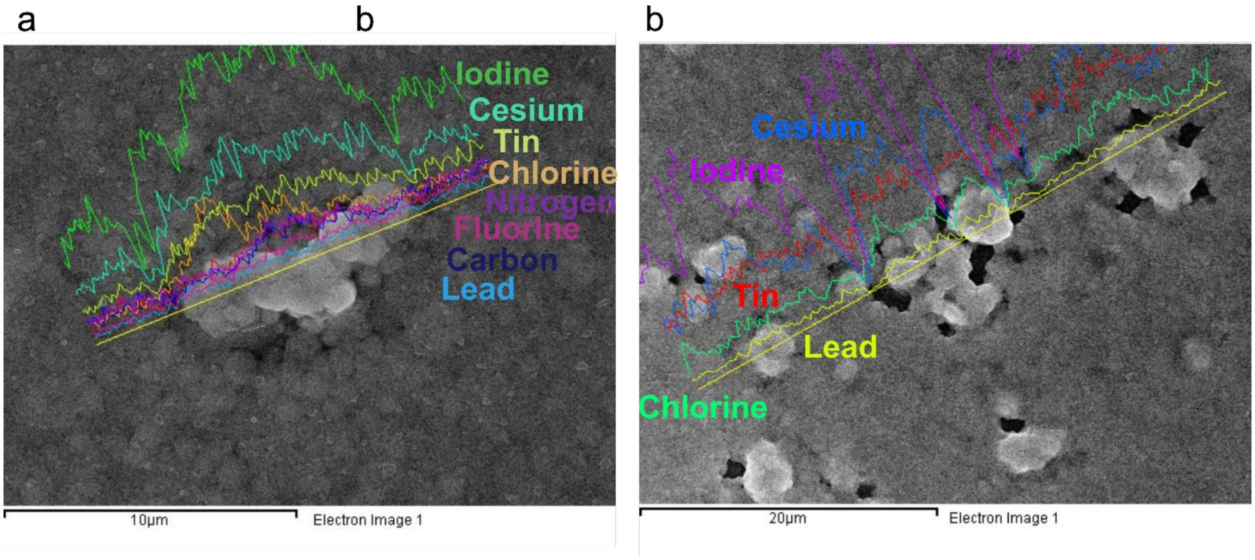

Fig. S 3. Line scan EDX analysis demonstrating the variation of each element across one of the brighter spots observed on top view SEM image of a Sn-Pb halide perovskite film processed with 45mol% of MACl. a. spot 1: Green - Iodine, Cyan - Cesium, Yellow - Tin, Orange - Chlorine, Purple - Nitrogen, Pink - Fluorine, Blue - Carbon, Light blue - Lead). b: spot 2: Pink - Iodine, Blue - Cesium, Red - Tin, Green - Chlorine, Yellow – Lead. The most abundant elements are Cs and I, supporting the formation of segregated CsI-rich phases.

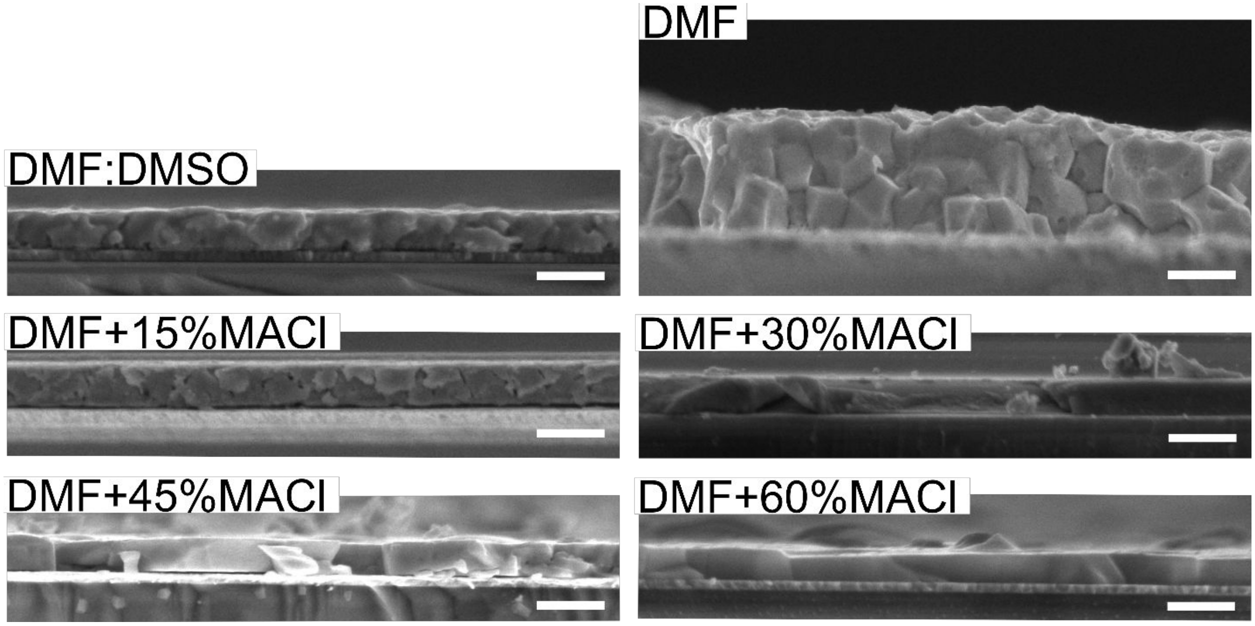

Fig. S 4. SEM cross sections of mixed Sn-Pb halide perovskite thin films processed with and without DMSO and different quantities of MACl within the precursor solution. Scale bar is 1 µm.

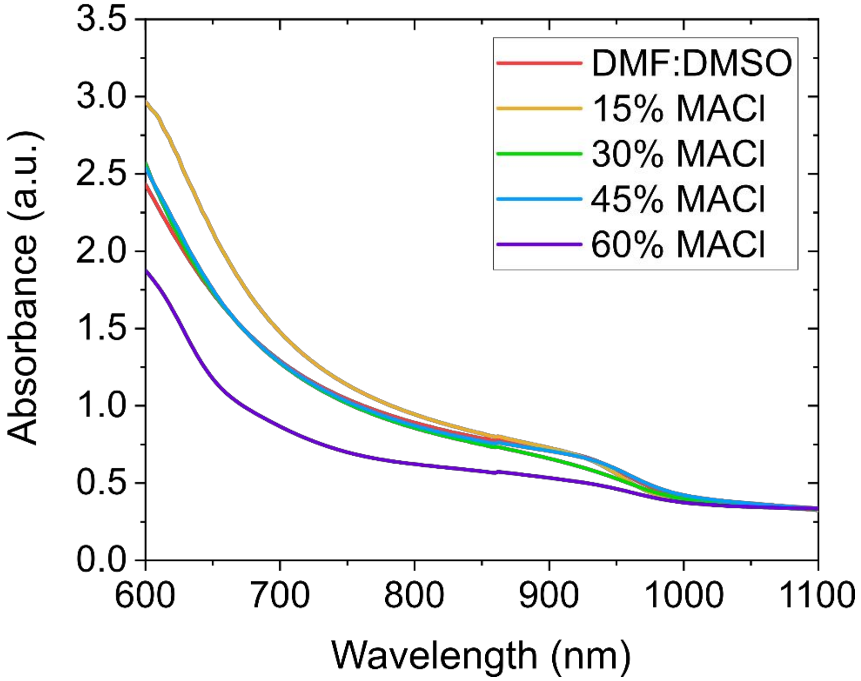

Fig. S 5. UV-Vis spectra of Sn-Pb halide perovskite thin films with and without DMSO and different quantities of MACl. The film containing 60 mol% MACl appears more transparent than the others, likely due to a high excess of organic cations within the material.

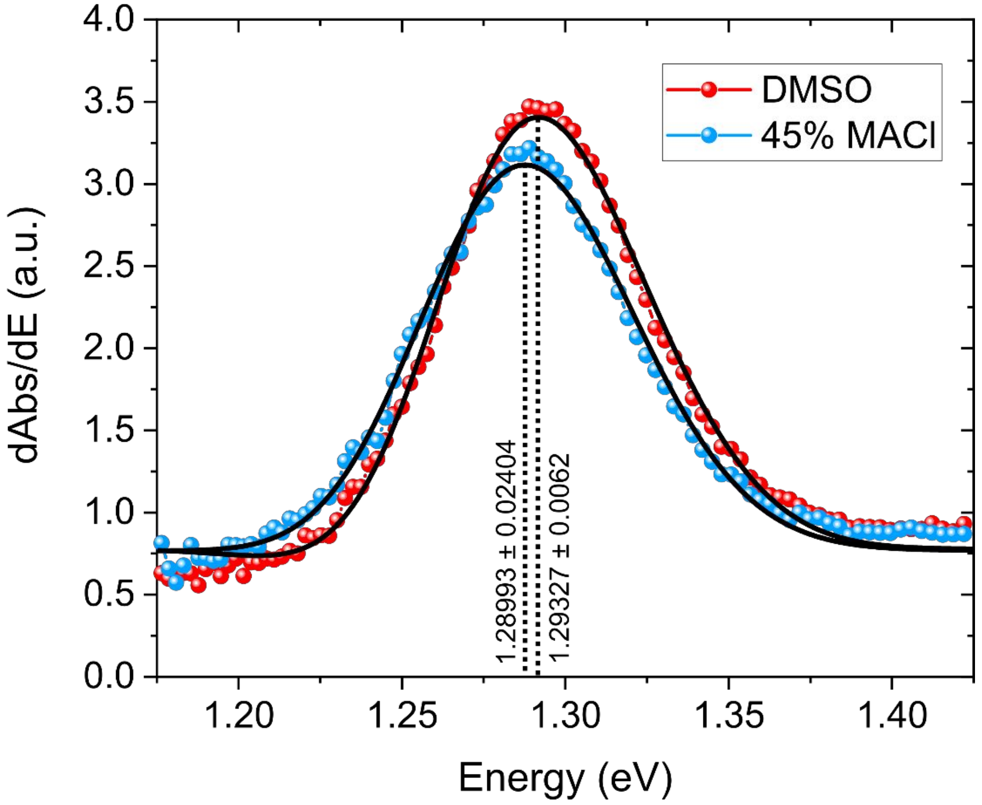

Fig. S 6. First derivative of the absorption spectrum of mixed Sn-Pb halide perovskite thin films processed with DMSO and without DMSO and 45 mol% MACl. The position of the peak maxima have been extracted through fitting.

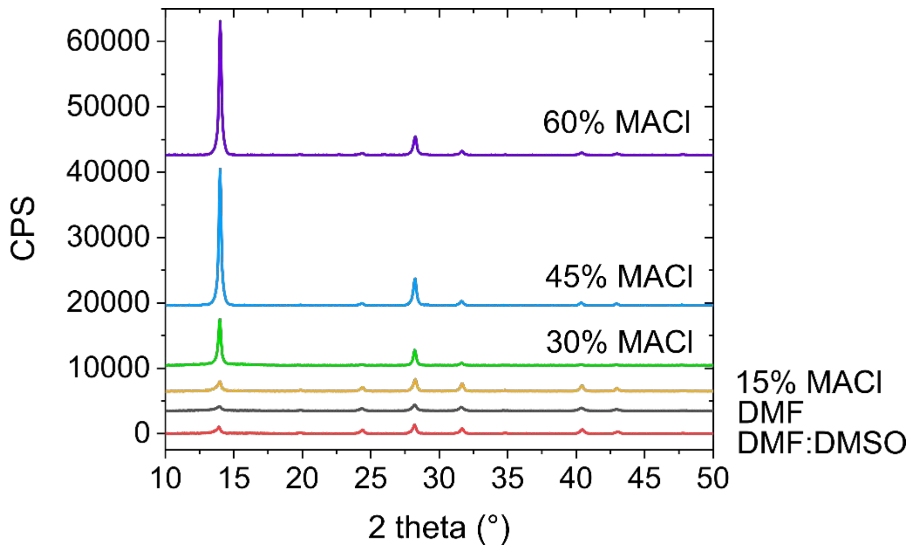

Fig. S 7. XRD patterns of mixed Sn-Pb halide perovskite thin films with and without DMSO and different quantities of MACl.

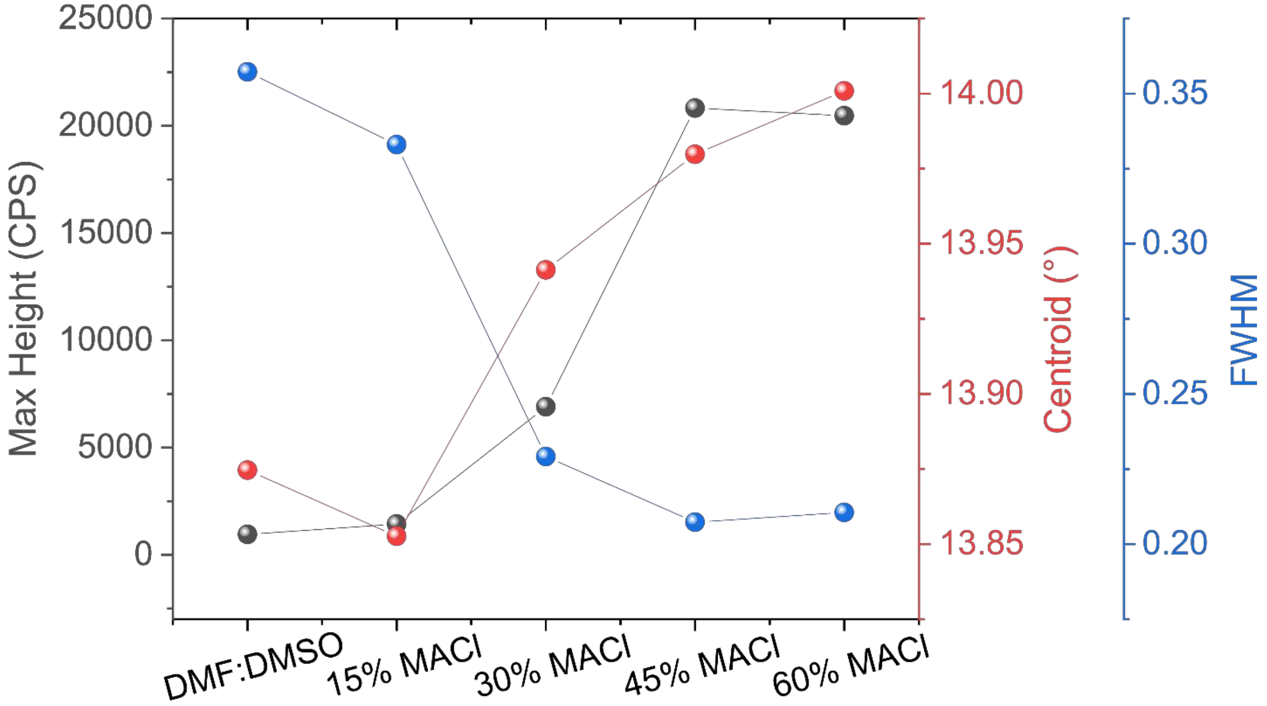

Fig.S 8. Maximum Height, centroid and FWHM of the (100) peak of mixed Sn-Pb halide perovskite thin films processed with and without DMSO and different quantities of MACl.

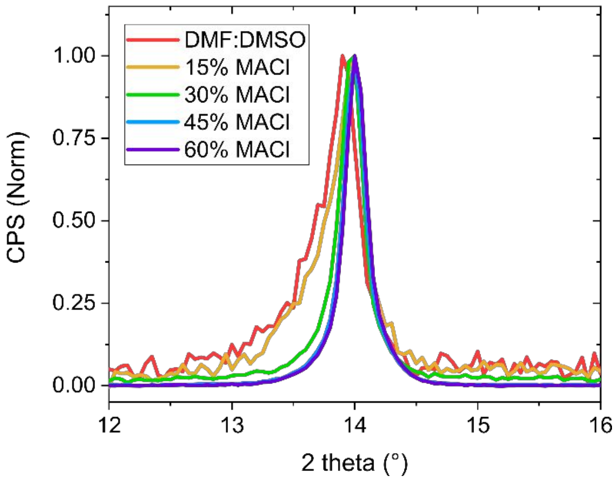

Fig. S 9. Magnified view of the normalized (100) peak in the XRD patterns of mixed Sn-Pb halide perovskite thin films processed with and without DMSO and different quantities of MACl.

Table S 1. Atomic concentrations of the main species in DMSO-containing and DMSO-free w MACl thin films derived from the XPS wide scan showed in Figure 2a.

	DMSO - Control	45% MACl
**O**	18.15	15.14
**C**	46.58	40.39
**N**	4.81	9.51
**Cs**	0.17	0.57
**Pb**	2.24	3.54
**Sn**	15.3	11.44
**F**	0.63	1.48
**I**	12.13	11.82
**Cl**	0	6.11

Table S 2. XPS fitting parameters: peak position, FWHM and relative concentration of the components corresponding to the decomposition of Sn $3d_{5/2}$, Cl 2p, N 1s, O 1s, C 1s core levels of the control DMSO-containing perovskite film and the DMSO-free perovskite treated with MACl.

		**Control DMSO-containing perovskite**			**DMSO-free, 45 mol% MACl perovskite**		
	**Name**	**Position (0.2 eV)**	**FWHM**	**%At Conc**	**Position**	**FWHM**	**%At Conc**
**N 1s**	**$C=NH_2^+$**	400.44	0.92	100	400.44	0.96	90.48
	**$C-NH_2$**				401.84	0.96	9.52

**O 1s**	**OH**	531.09	1.25	14.39	531.07	1.25	18.61
	**O=C**	532.12	1.25	35.73	532.1	1.25	42.13
	**O-C**	533.14	1.25	5.09	533.12	1.25	8.22
	**SnO_2; O-Sn-Cl**	530.51	1.17	44.8	530.69	1.02	31.04
**C 1s**	**C-C**	284.81	1.11	75.17	284.81	1.15	67.58
	**C=NH2+**	288.3	1.11	10.51	288.3	1.15	16.41
	**O-C=O**	289.51	1.11	3.94	289.51	1.15	3.78
	**C-O;C-N**	286.66	1.11	3.72	286.66	1.15	5.76
	**C-C-O**	285.85	1.11	6.67	285.93	1.15	6.46
**Sn $3d_{5/2}$**	**Sn(II)**	486.42	0.85	21.35	486.36	0.89	16.83
	**Surface Sn-phase**	486.83	1.33	78.65	487.06	1.30	83.17

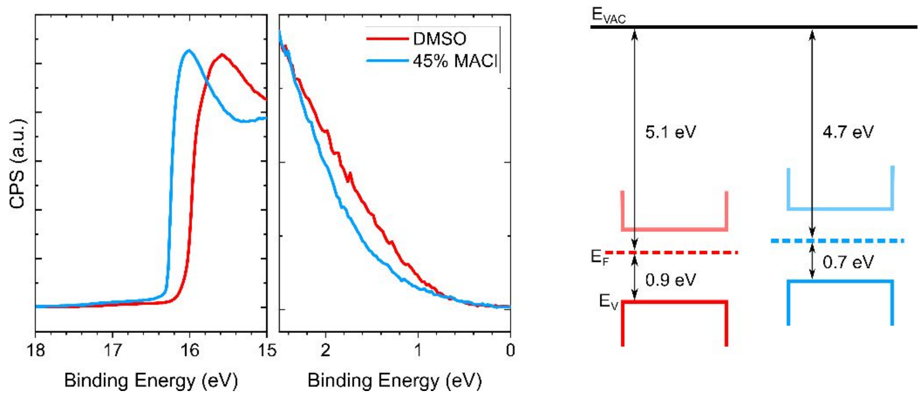

Fig. S 10. Valence band edge position with respect to the Fermi level (corresponding to the zero of the binding energy – BE – scale) obtained from UPS spectra and calibrated with the XPS measurement of the valence band for the control DMSO-containing film (red) and DMSO-free MACl treated thin film (light blue); Energy level diagram obtained from UPS fitting. The energy bandgap has been obtained by UVVis (see figure S6). Dashed lines indicate the position of the Fermi level.

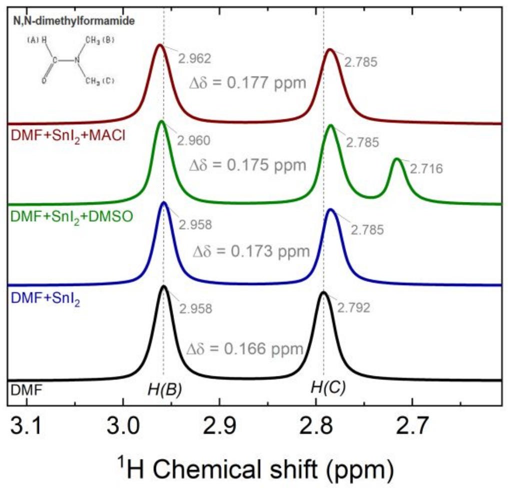

Fig. S 11. ^{1}H NMR spectra of DMF, SnI_2 in DMF, SnI_2 in DMF:DMSO and SnI_2+MACl in DMF. the spectra were aligned using the formyl proton (H(A)) of DMF at 8.03 ppm. In the presence of SnI_2, the N-methyl protons (H(C)) of DMF shifts slightly upfield, indicating coordination between DMF and SnI_2. Upon addition of DMSO, the H(C) signal of DMF does not shift, while the N-methyl protons (H(B)) signal shifts downfield. When DMSO is replaced by MACl, the N-methyl protons (H(B)) signal shifts even more strongly, further increasing the separation between the two methyl peaks of DMF (Δ(δ)).

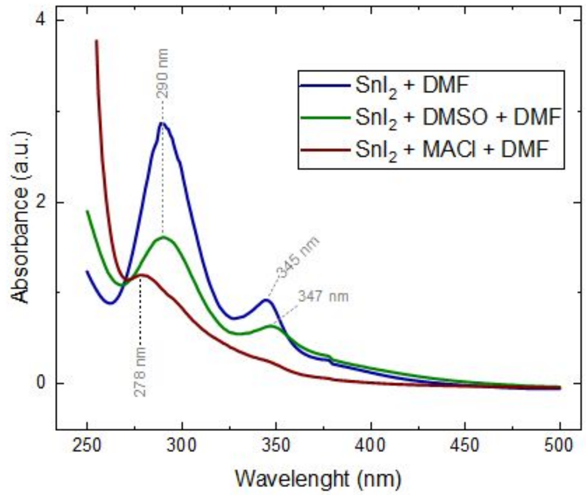

Fig. S 12. UV-Vis absorption spectra of SnI_2 in DMF, SnI_2 in DMF:DMSO and SnI_2+MACl in DMF , diluted in acetonitrile to avoid saturation of the detector. Both DMF and DMF:DMSO systems exhibit the presence of Sn^{4+} species, clearly showing typical SnI_4 peaks at about 350 nm and 290 nm.[10] The addition of MACl considerably suppresses the presence of Sn^{4+} in solution, suggesting that MA^+/Cl^- already interacts with SnI_2 in solution.

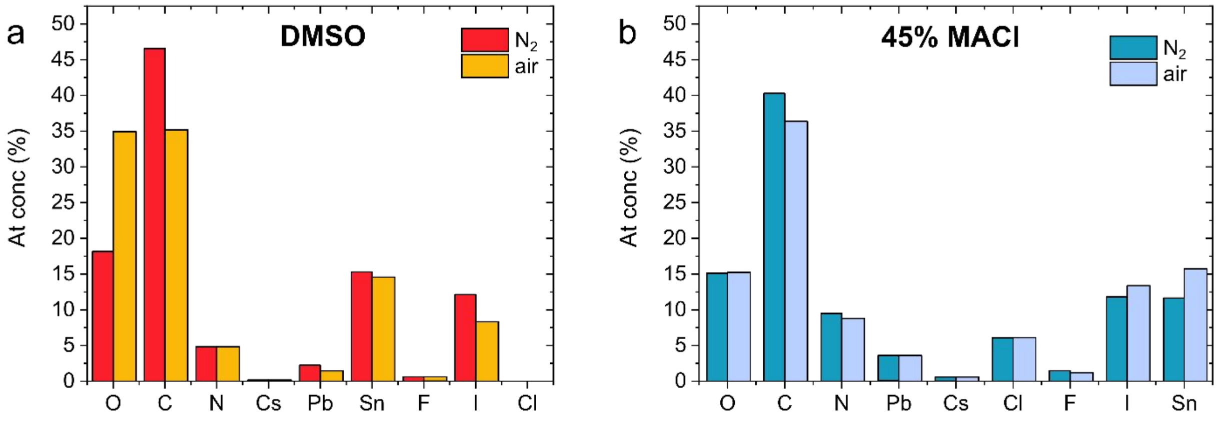

Fig. S 13. Atomic quantification of a. control DMSO-containing film and b. DMSO-free MACl treated thin film before and after oxidation induced by ambient atmosphere exposure for 2 days.

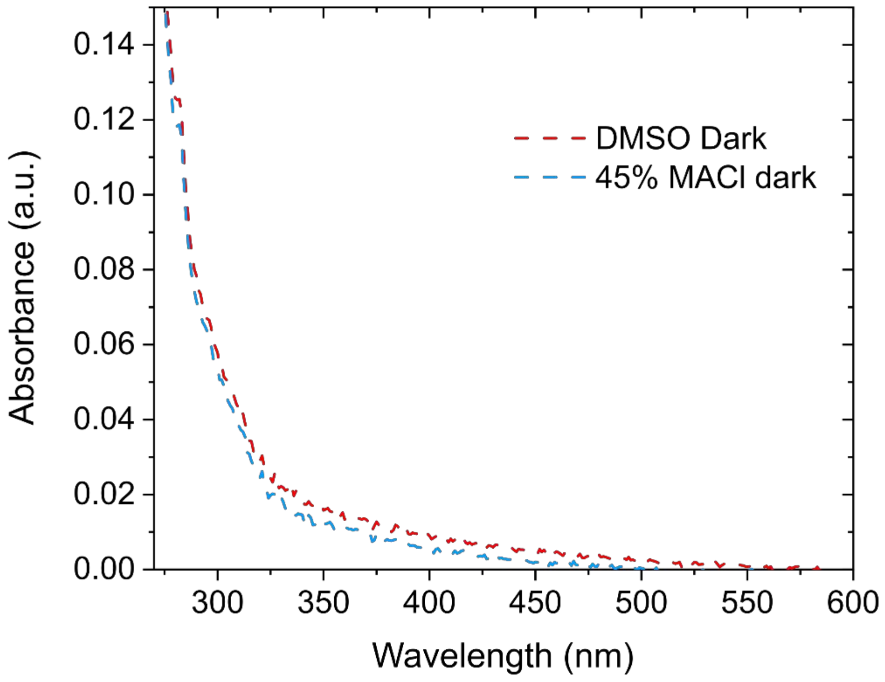

Fig. S 14. UV-Vis spectroscopy of the hexane solution where the control DMSO-containing sample and MACl-treated sample have been immersed in for 100 hours in the dark.

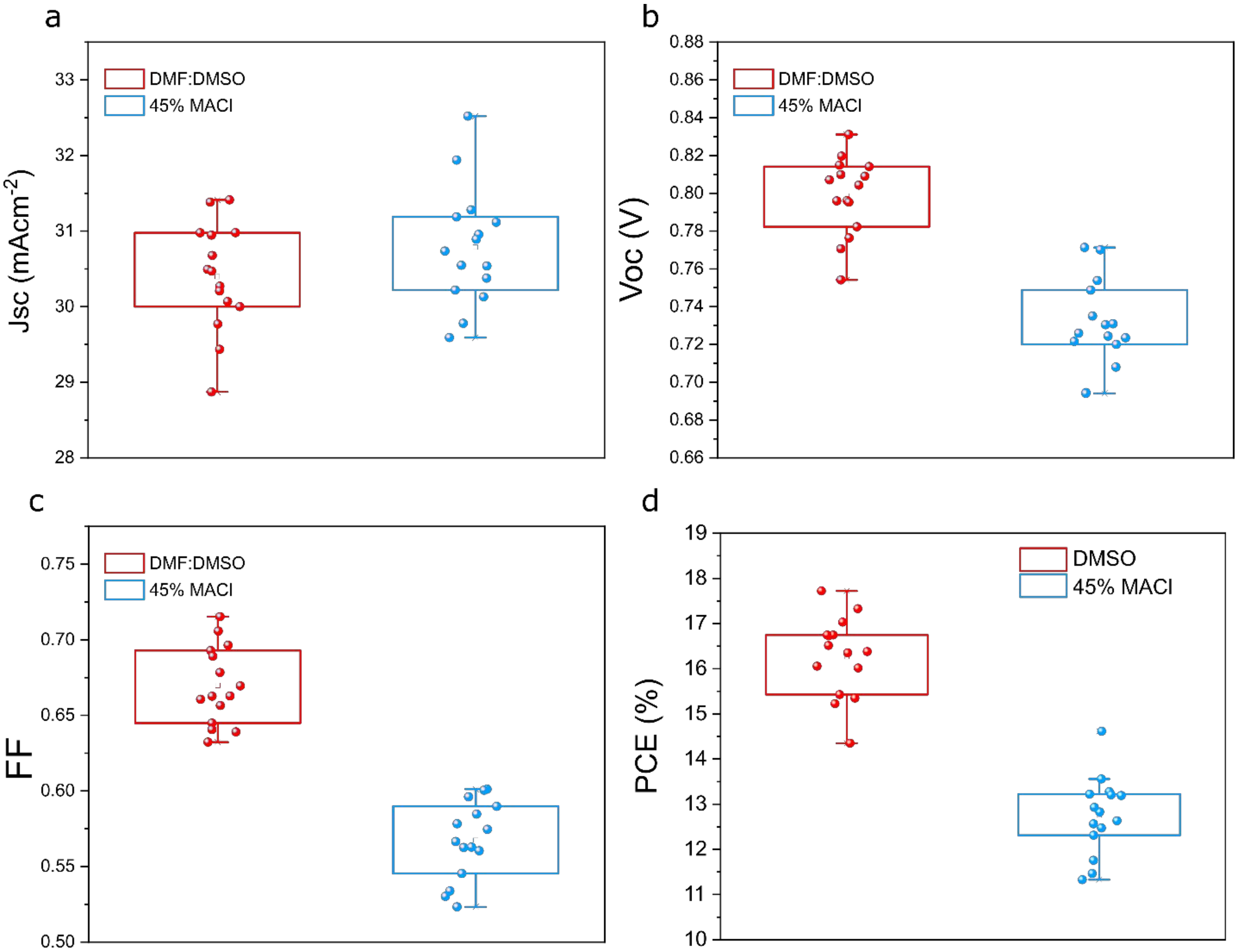

Fig. S 15. Box plots of the short circuit current, open circuit voltage, fill factor and power conversion efficiency of the control DMSO-containing perovskite solar cells and devices fabricated without DMSO and with 45mol% MACl.

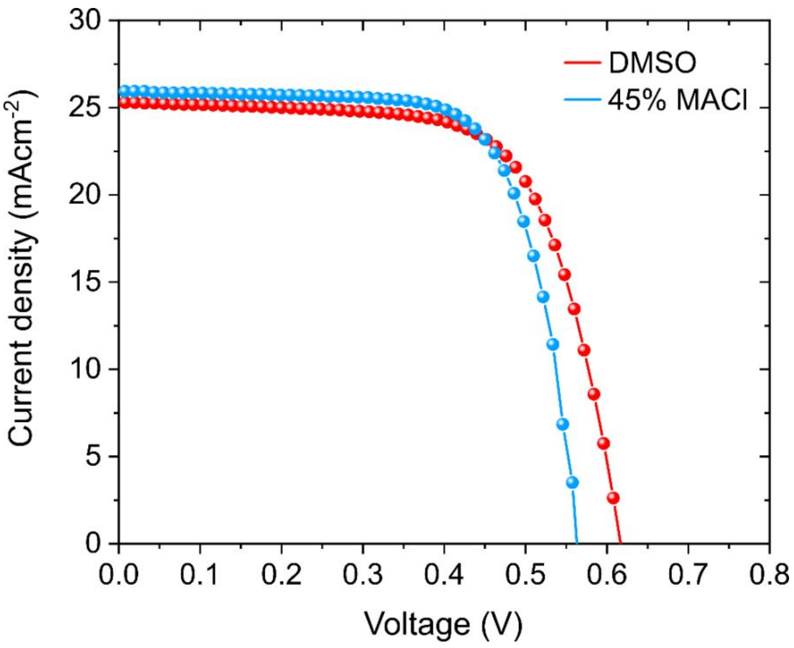

Fig. S 16. JV curves of representative as-deposited control DMSO and MACl-containing cells used for the MPP stability test.

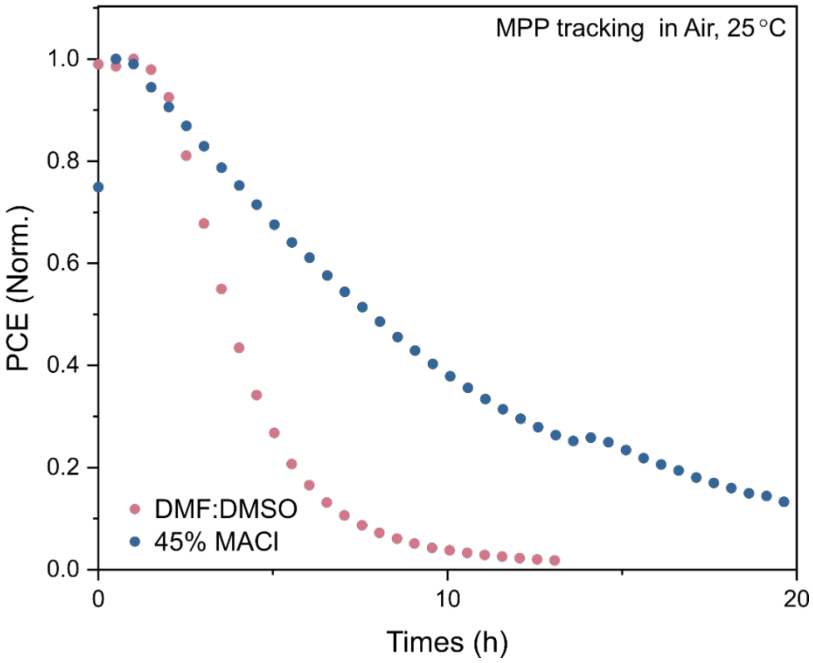

Fig. S 17. MPPT stability tests of control and MACl-treated devices. Solar cells have been aged at 25°C, in ambient atmosphere, without encapsulation under continuous MMPT and simulated 1 sun illumination.

References

(1) Fairley, N.; Fernandez, V.; Richard-Plouet, M.; Guillot-Deudon, C.; Walton, J.; Smith, E.; Flahaut, D.; Greiner, M.; Biesinger, M.; Tougaard, S.; Morgan, D.; Baltrusaitis, J. Systematic and Collaborative Approach to Problem Solving Using X-Ray Photoelectron Spectroscopy. *Applied Surface Science Advances* 2021, *5* (100112), 100112. https://doi.org/10.1016/j.apsadv.2021.100112.

(2) Huerta Hernandez, L.; Haque, M. A.; Sharma, A.; Lanzetta, L.; Bertrandie, J.; Yazmaciyan, A.; Troughton, J.; Baran, D. The Role of A-Site Composition in the Photostability of Tin–Lead Perovskite Solar Cells. *Sustainable Energy Fuels* 2022, *6* (20), 4605–4613. https://doi.org/10.1039/D2SE00663D.

(3) Helander, M. G.; Greiner, M. T.; Wang, Z. B.; Lu, Z. H. Pitfalls in Measuring Work Function Using Photoelectron Spectroscopy. *Appl Surf Sci* 2010, *256* (8), 2602–2605. https://doi.org/10.1016/j.apsusc.2009.11.002.

(4) Giannozzi, P.; Baroni, S.; Bonini, N.; Calandra, M.; Car, R.; Cavazzoni, C.; Ceresoli, D.; Chiarotti, G. L.; Cococcioni, M.; Dabo, I.; Corso, A. D.; de Gironcoli, S.; Fabris, S.; Fratesi, G.; Gebauer, R.; Gerstmann, U.; Gougoussis, C.; Kokalj, A.; Lazzeri, M.; Martin-Samos, L.; Marzari, N.; Mauri, F.; Mazzarello, R.; Paolini, S.; Pasquarello, A.; Paulatto, L.; Sbraccia, C.; Scandolo, S.; Sclauzero, G.; Seitsonen, A. P.; Smogunov, A.; Umari, P.; Wentzcovitch, R. M. QUANTUM ESPRESSO: A Modular and Open-Source Software Project for Quantum of Materials. *Journal of Physics: Condensed Matter* 2009, *21* (39), 395502. https://doi.org/10.1088/0953-8984/21/39/395502.

(5) van Setten, M. J.; Giantomassi, M.; Bousquet, E.; Verstraete, M. J.; Hamann, D. R.; Gonze, X.; Rignanese, G. M. The PSEUDODOJO: Training and Grading a 85 Element Optimized Norm-Conserving Pseudopotential Table. *Comput Phys Commun* 2018, *226*, 39–54. https://doi.org/10.1016/j.cpc.2018.01.012.

(6) Adamo, C.; Barone, V. Toward Reliable Density Functional Methods without Adjustable Parameters: The PBE0 Model. *J Chem Phys* 1999, *110* (13), 6158–6170. https://doi.org/10.1063/1.478522.

(7) Kubicki, D. J.; Prochowicz, D.; Hofstetter, A.; Zakeeruddin, S. M.; Grätzel, M.; Emsley, L. Phase Segregation in Cs-, Rb- and K-Doped Mixed-Cation (MA)x(FA)1-XPbI3 Hybrid Perovskites from Solid-State NMR. *J Am Chem Soc* 2017, *139* (40), 14173–14180. https://doi.org/10.1021/jacs.7b07223.

(8) Zong, Y.; Wang, N.; Zhang, L.; Ju, M.; Zeng, X. C.; Sun, X. W.; Zhou, Y.; Padture, N. P. Homogenous Alloys of Formamidinium Lead Triiodide and Cesium Tin Triiodide for Efficient Ideal-Bandgap Perovskite Solar Cells. *Angewandte Chemie* 2017, *129* (41), 12832–12836. https://doi.org/10.1002/ange.201705965.

(9) Li, S.; Jiang, Y.; Xu, J.; Wang, D.; Ding, Z.; Zhu, T.; Chen, B.; Yang, Y.; Wei, M.; Guo, R.; Hou, Y.; Chen, Y.; Sun, C.; Wei, K.; Qaid, S. M. H.; Lu, H.; Tan, H.; Di, D.; Chen, J.; Grätzel, M.; Sargent, E. H.; Yuan, M. High-Efficiency and Thermally Stable FACsPbI3 Perovskite Photovoltaics. *Nature* 2024, *635*, 82–88. https://doi.org/10.1038/s41586-024-08103-7.

(10) Treglia, A.; Ambrosio, F.; Martani, S.; Folpini, G.; Barker, A. J.; Albaqami, M. D.; De Angelis, F.; Poli, I.; Petrozza, A. Effect of Electronic Doping and Traps on Carrier Dynamics in Tin Halide Perovskites. *Mater Horiz* 2022, *9*, 1763–1773. https://doi.org/10.1039/D2MH00008C.
